# Frequency of Gene Polymorphisms in Admixed Venezuelan Women with Recurrent Pregnancy Loss: Microsomal Epoxy Hydroxylase (rs1051740) and Enos (rs1799983)

**DOI:** 10.3390/cimb46040217

**Published:** 2024-04-17

**Authors:** María Johanna Peña, Claudia Valentina De Sanctis, Juan Bautista De Sanctis, Jenny Valentina Garmendia

**Affiliations:** 1 Institute of Immunology, Faculty of Medicine, Universidad Central de Venezuela, Caracas 1040, Venezuela; mariajohanna.sanoja@gmail.com (M.J.P.); claudiavdesanctis@gmail.com (C.V.D.S.); 2 Institute of Molecular and Translational Medicine, Faculty of Medicine and Dentistry, Palacky University, 779 00 Olomouc, Czech Republic

**Keywords:** recurrent pregnancy loss, single nucleotide gene polymorphism (SNP), microsomal epoxy hydrolase (mEPH), endothelial nitric oxide synthase (eNOS), rs1051740, rs1799983

## Abstract

Recurrent pregnancy loss (RPL) affects around 2% of women of reproductive age. Primary RPL is defined by ≥2 pregnancy losses and no normal birth delivery. In secondary RPL, the losses are after a normal pregnancy and delivery. Most cases have no clear aetiology, although primary cases are the most complex. Several gene single nucleotide polymorphisms (SNPs) have been associated with RPL. The frequency of some SNPs is increased in women suffering from RLP from Asian or Caucasian races; however, in admixed populations, the information on possible genetic links is scarce and contradictory. This study aimed to assess the frequency of two SNPs present in two different enzymes involved in medical conditions observed during pregnancy. It is a case–control study. Microsomal epoxy hydrolase (mEPH) is involved in detoxifying xenobiotics, is present in the ovaries, and is hormonally regulated. The endothelial nitric oxide synthase (NOS3) that forms nitric is involved in vascular tone. Two SNPs, rs1051740 (mEPH) and rs1799983 (NOS3), were assessed. The study included 50 controls and 63 primary RPL patients. The frequency of mutated alleles in both SNPs was significantly higher in patients (*p* < 0.05). Double-mutated homozygotes were encountered only in RPL patients (*p* < 0.05). Genetic polymorphisms rs1051740 and rs1799983 may be involved in primary RPL in the Venezuelan admix population. Genetic studies could provide crucial information on the aetiology of primary RPL.

## 1. Introduction

Oxidative stress is a term used to designate an imbalance between the production of reactive oxygen species (ROS) and antioxidant levels; the production of ROS exceeds the quantity of antioxidants needed to neutralise it [1,2,3]. The ROS family includes superoxide, hydroxy radical, peroxide radical, radical alkoxyl, hydroperoxyl radical, and non-intermediary radicals (hydrogen peroxide, hypochlorite acid, nitrogen peroxide, and peroxynitrite). These compounds are unstable due to their free electrons [1,2,3]. There are multiple sources of ROS production, such as cellular respiration, inflammatory responses, metabolism of drugs and xenobiotics, exposure to ionising radiation (RX and UV), monoamine oxidase activity, purine catabolism, uric acid production, catalytic reactions supported by different enzymes (xanthine oxidase, NADPH oxidase, nitric oxide synthase, and heme oxidase), nitrogen peroxide formation, and protein synthesis in the rough endoplasmic reticulum (RER) [4,5,6].

The body has enzymatic and non-enzymatic antioxidant mechanisms. Most non-enzymatic mechanisms are obtained from exogenous sources (vitamin C and E), and the rest are synthesised in the body, such as transferrin, ceruloplasmin, and the glutathione reduction system (tripeptide, L-glutamate, L-cysteine, and glycine). Among all the antioxidant mechanisms, superoxide dismutase, peroxidases, catalase, and thioredoxin are extremely valuable [4,5,6].

ROS production during pregnancy and its medical complications, including RPL, have been studied [7,8,9]. ROS production in a healthy placenta increases due to new tissue formation and increased metabolic rate, but higher production and availability of antioxidant enzymes and metabolites compensate for it [8,9]. Increased levels of oxidative stress are associated with several gestational pathologies, such as gestational diabetes, miscarriage, recurrent pregnancy loss, defects during embryogenesis, the formation of teratomas, slowed intrauterine growth, and preeclampsia [10,11,12,13,14,15,16]. Excessive ROS production and insufficient antioxidants can kill the embryo [8,9,10,11,12,13,14]. Therefore, antioxidant uptake and production are upregulated during pregnancy. The body produces higher catalase levels, glutathione peroxidase, and Cu/Zn or Mn superoxide dismutase [11,16,17]. Genetic associations with detoxifying enzymes and other proteins involved in inflammation and radical elimination were reported in both normal and medical complications during pregnancy [18,19]. However, most analyses were performed in Caucasian and Asian populations, and brief reports in admixed populations were contradictory [19].

Epoxy hydrolases are critical enzymes that transform epoxide-containing lipids (modified by ROS) by adding water [20]. At least four enzymes catalyse the reaction; however, the most important ones are the microsomal, mEPH or EPHX1, and the soluble one, EPHX2 [21]. Both enzymes have been involved in cardiovascular diseases and other medical conditions [20,21]. In addition, gene variants of EPHX1, particularly rs1051740, have been shown to affect enzyme activity [20,21]. The decrease in enzyme activity may involve several processes, including female sexual hormonal regulation and endometrial tissue [21]. It is currently unknown whether gene polymorphisms in the mEPH gene play a role in RPL or if there are possible associations with other polymorphisms.

Nitric oxide metabolites in pregnancy and pregnancy complications have been extensively studied [22,23], including RPL [24,25,26,27,28]. A possible association between endothelial nitric oxide synthase and RPL has been suggested, especially in exon 8, rs1799983 (+894G>T). Nonetheless, questions remain about the impact of polymorphism on tissue, its association with specific races or populations, and its possible relation to other genes [25,26,27,28]. It is important to note that admixed populations are particular and involve different gene segregations. The admixed Venezuelan population differs from other Latin American populations [29,30].

Several analyses of nitric oxide metabolites and synthase enzymes in plasma, serum, and placenta have been controversial. Alemán and colleagues [31] in Vargas Hospital, Caracas, reported an increased amount of nitrite serum levels in preeclampsia patients compared to healthy pregnant women, as well as increased expression of NOS enzymes (eNOS and iNOS) in the placenta of these patients compared to controls. These results differ from other reports independently of the ethnic difference [32,33]. Our group [34] demonstrated, as have different authors [32,33], a significant reduction in serum levels of nitric oxide products (nitrites and nitrates) in preeclamptic patients compared to a control group [34].

Even though genetic polymorphism provides partial information on disease mechanisms, the increased frequency of the homozygous mutation, which corresponds to impaired enzyme function, may be useful. Both enzymes, mEPH and NOS3, have been linked to medical problems during advanced pregnancy, not in early pregnancy or implantation. Since those processes are very difficult to study in human pregnancy, genetic polymorphism analysis can probably provide useful information on the involvement of both enzymes in RPL.

The present study aimed to evaluate certain aspects of oxidative metabolism in patients with frequent miscarriages by determining genetic polymorphisms in microsomal epoxy hydroxylase (mEPH, T612C, rs1051740) and nitric oxide synthase 3 (eNOS. G894T, rs1799983) in admixed Venezuelan women with primary RPL.

## 2. Materials and Methods

The study is a case–control study. It involved 63 patients with criteria for primary RPL. Patients with RPL were screened for immunological and hormonal factors and had no alterations in paraclinical analysis (no infectious or genetic diseases). Patients with secondary RPL, patients with cancer, severe endometriosis, and HPV infection were excluded. All patients suffered from primary RPL. The study included 50 healthy women with normal pregnancies and no medical conditions (no viral disease, hypertension, diabetes, metabolic syndrome, or hormonal disbalances). Written consent was obtained from all individuals interested in the study. The genetically admixed characteristics of patients and controls were certified as described before [30]. The Ethical Committee of the Institute of Immunology, Faculty of Medicine, Caracas, Venezuela, approved the study (number 20052308).

Ten mL of venous blood was collected in EDTA tubes from all individuals, and the cDNA was extracted from the buffy coat the same day. Genomic DNA isolation was performed following the instructions of a commercial kit: the AxyPrep Blood Genomic DNA Miniprep Kit (Axygen Biosciences, Union City, CA, USA). This kit can purify up to 12 µg of genomic DNA in 250 µL of non-coagulated blood. Briefly, 500 µL of AP1 lysis buffet was mixed with 200 µL of buffy coat in a 1.5 mL Eppendorf tube by vortexing for 10 s. Then, 100 µL of AP2 buffer was added and vortexed for 20 s to encourage protein precipitation before centrifuging for 10 min at 4000× *g* at room temperature. The supernatant was added to the AxyPrep column. Then, the column was centrifuged for 2 min at 2000× *g*. The eluate was discarded, and the column was loaded again with 700 µL of W1A washing buffer and centrifuged at 2000× *g* for 1 min. The eluate was discarded, and the column was loaded with W2 washing buffer and centrifuged at 4000× *g* for 1 min. This step was repeated with the W2 washing buffer. The column was then placed in a 1.5 mL Eppendorf tube, 200 µL of TE elution buffer was added, and after incubating it for 5 min, the tube was centrifuged at 4000× *g* for 1 min to obtain genomic DNA.

DNA concentration was calculated using the spectrophotometer Gene Quant II for DNA/RNA (Amersham Pharmacy Biotech^®^ calculator, Piscataway, NJ, USA). The purity of the DNA was checked using the standard 260 nm and 280 nm for nucleotides and proteins, respectively. The ratio 260/280 of the samples was ≥1.8. The required DNA concentration was adjusted using nuclease-free water (Promega^®^, Madison, WI, USA). The DNA concentration was expressed in µg/mL and was automatically calculated by the spectrophotometer. Purified DNA was stored at −20 °C until needed.

The DNA was amplified via PCR using the following primers:

mEPH forward 5′ GATCGATAAGTTCCGTTTCACC 3′
reverse 5′ ATCCTTAGTCTTGAAGTGAGGAT 3′
eNOS, forward 5′ AAGGCAGGAGACAGTGGATG 3′
reverse 5′ CAGTCAATCCCTTTGGTGCT 3′.

For the analysis of the SNP rs1051740 (mEPH), Cheng S.L. et al.’s protocol was used [35]. Briefly, DNA (20 ng) was mixed with a 40 µL reaction mixture containing 1.5 mM MgCl_2_, 100 ng of each primer, 500 µM deoxyribonucleoside triphosphate, and 0.6 IU Taq DNA polymerase (Promega^®^). The DNA was amplified via polymerase chain reaction (PCR) using a thermal cycler Minicycler™ (MJ Research, Waltham, MA, USA) consisting of an initial single cycle of 10 min at 95 °C, followed by 35 cycles of 30 s at 94 °C, 20 s at 52 °C, and 5 s at 72 °C. Then, PCR products were treated with *Eco* RV (Promega^®^). The digested samples were then loaded on an ethidium bromide-stained 2.5% agarose gel, and the products were visualised using UV transillumination. RFLP products are for wild homozygous TT genotypes: two bands: 140 pb/22 pb; for heterozygous CT: 162 pb/140 pb/22 pb; and for the mutated homozygous, CC 162 pb. The molecular weight marker was the *Hae* III digested ΦX174 (Promega^®^).

The protocol described by Leeson et al. [36] was used for the analysis of the rs1799983 SNP with minor modifications. The sample of genomic DNA, 50 ng, was added to a mixture of 50 mmol/L KCl, 10 mmol/L Tris (pH 8.3), 0.2 mmol/L of each dNTP, 10 nmol of each primer, and 2U of Taq DNA polymerase (Promega^®^), for a total volume of 30 µL. The amplification was carried out using the Minicycler™. The protocol was denaturation at 95 °C for 10 min, followed by 35 cycles of 30 s at 63 °C and 45 s at 72 °C. Ten microliters of the PCR products were digested with 2U *Dpn II* (Promega^®^), which cuts when the T allele is present at position 894 (corresponding to Asp298). The RFLP products were GG: wild homozygous 248 pb; GT: heterozygous 248 pb/160 pb/88 pb; and TT: mutated homozygous 160 pb/88 pb. The RFLP products were visualised and analysed as described before.

All the amplifications were sequenced by the sequence service at the Venezuelan Institute for Scientific Research (IVIC). All the results of the RFLP were confirmed.

### Statistical Analysis of Results

The minimum sample size was set at 45. The calculation was performed using the online calculator (www.calculator.net, accessed on 2 April 2024) based on the SNP data of 3% frequency in the normal population for rs1051740 (https://opensnp.org, accessed on 2 April 2024).

The program GraphPad Prism 5 was used for calculations. The results were analysed using chi-square with Yate’s correction. Both absolute and relative frequency have been utilised, and significance has been attributed to a *p*-value less than 0.05.

## 3. Results

The general data of the women who participated in the study are represented in Table 1. The group of controls was from the same area as the patients. As compared to controls, patients have a significantly lower pregnancy duration (*p* < 0.001) and a higher number of pregnancy complications (*p* < 0.05).

**Figure 1:**
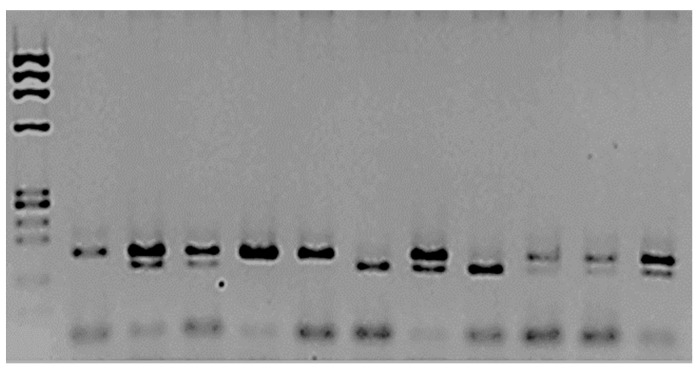
Analysis of mEPH polymorphism rs1051740.

Figure 1 illustrates the SNP analysis of mEPH. The frequency of the T allele in mEPH was 0.86 for controls and 0.6 for patients (*p* = 0.1). On the other hand, the allelic frequency of the C allele was 0.14 for controls and 0.4 for patients; the difference was significant (*p* = 0.04).

The amplified and digested PCR products were separated using 2.5% agarose. The first column corresponds to the MW marker ΦX174/*Hae* III. The second is the amplified product, which was not treated with *Eco RV* enzyme; two bands, 162 bp and 22 bp, were observed. The rest of the samples were treated with the restriction enzyme. Columns 3, 4, 8, and 12 correspond to heterozygotes 162/142/22 bp. Columns 5, 10, and 11 correspond to the two bands (wt TT): 140 bp/22 bp. The mutated homozygous are columns 7 and 9, CC 162 bp.

**Figure 2:**
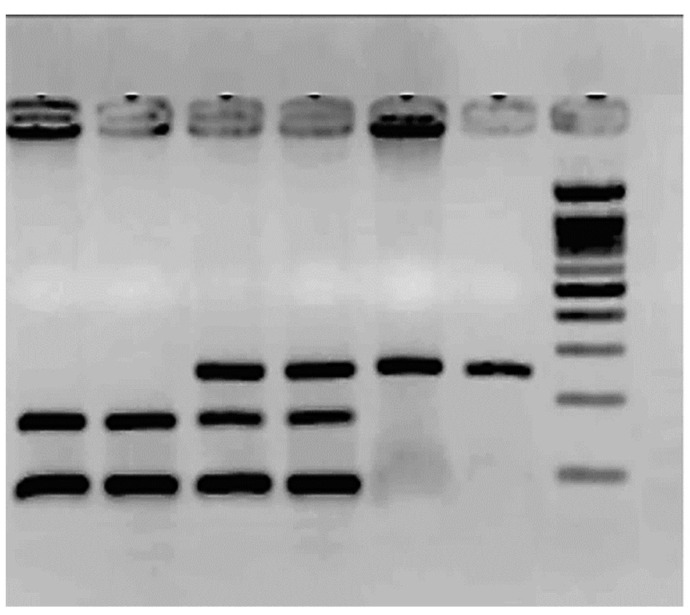
Agarose gel electrophoresis (2.5%) for the SNP rs1799983 polymorphism.

Figure 2 shows the results of the SNP rs1799983. The frequency of the G allele was 0.84 in controls and 0.7 in patients for the T allele (*p* = 0.7). The T allele frequency is 0.16 in controls and 0.3 in patients (*p* = 0.2). The TT homozygote was only observed in six patients; see Table 2. There is a high number of heterozygotes in the control group.

**Table 2:** The frequencies of rs1799983 (eNOS, exon 3) are presented.

	Controls (n = 40)	Patients (n = 63)	*p*
Glu298Glu	34 (68%)	34 (54%)	0.53
Glu298Asp	16 (32%)	23 (36.5%)	0.85
Asp298Asp	0 (0%)	6 (9.5%)	**0.04**

The molecular weight ladder (ΦX174/*Hae* III) is the last column. The rest corresponds to PCR products treated with the enzyme *Dpn* *II*. Columns 1 and 2 correspond to two TT: mutated homozygous 160 bp/88 bp. Columns 3 and 4 correspond to GT heterozygous 248 bp/160 bp/88 bp, and columns 5 and 6 to GG wild-type homozygous, 248 bp.

Table 3 displays the results of the genetic polymorphism of mEPH. There were significant differences (*p* < 0.05) between controls and patients in normal and mutated homozygotes.

**Table 3:** The frequencies of rs1051740 (mEPH, exon 8) are represented.

	Controls (n = 50)	Patients (n = 63)	*p*
Tyr113Tyr	37 (74%)	30 (47.6%)	0.17
Tyr113His	12 (24%)	23 (36.5%)	0.33
His113His	1 (2%)	10 (15.9%)	**0.03**

The homozygous mutation was only observed in patients and was found to be statistically significant (*p* < 0.05). However, the mutated T allele frequency was not substantial (*p* = 0.4), likely due to the high number of heterozygotes in the control group.

Table 4 represents both polymorphisms. It was observed that there is a significant difference between patients and the control group when both polymorphisms are present. In the control group, only one individual has the mutated allele for mEPH and is a heterozygote for eNOS. However, four patients have the mutated gene mEPH and are heterozygotes for eNOS. Patients’ probability of having double-mutated alleles is significantly higher (*p* < 0.05). The number of heterozygotes for both genes was similar in the two groups; however, the number of homozygotes with wild-type genes was lower in patients than in controls, although it was insignificant.

## 4. Discussion

In the present study, a higher frequency of each SNP polymorphism, rs1051740 and rs1799983, was observed in patients with primary RPL. Interestingly, the haplotype analysis revealed the presence of both polymorphisms in RPL patients. These simple and combined analyses suggest that lower or unfunctional enzyme activities may be associated with RPL. This is the first report on rs1051740 in primary RPL patients and on both rs1051740 and rs1799983 in the Venezuelan admixed population certified upon HLA genetic patterns. The Venezuelan admixed population markedly differs from other admixed populations in Latin America [30].

Microsomal epoxy hydroxylase (EPHX1. EC 3.3.2.9.0) is a highly conserved enzyme that conjugates the toxic products of trans dihydro diol and epoxides to be inert products excreted from the organism. Humans gave two EPHX enzymes: microsomal EPHX (EPHX1) and soluble EPHX (EPHX2). EPHX1 mutations have been associated with many types of cancer, including preeclampsia and hypercholesterolemia or increased serum concentration of bile acid [36,37]. Certain genetic variations of soluble epoxy hydrolase (EPHX2) are responsible for increased oxidative metabolism and the inactivation of epoxyeicosatrienoic acids (EETs), which play a role in vascularisation. The decrease in enzyme activity of EPHX2 has been involved in hypertension [38] and preeclampsia [39,40,41]. The similarities between both enzymes, EPHX1 and EPHX2, suggest similar regulation and possible biological compensation [20,21,42]. The role of both enzymes and their polymorphisms in RPL should be carefully analysed.

In the menstrual cycle, EPHX1 is regulated in the endometrium by progesterone and is involved in oestrogen production [43], but its role in implantation is less known [44]. In this study, we observed that the frequency of the polymorphic variant His113His (rs1051740) of the mEPH was higher in patients than in controls. As reported, the genotype has been associated with lower enzymatic activity [20,21], possibly related to a higher incidence of implantation failure and vascular complications in pregnancy. This is the first report of mEPH in RPL and an admixed population.

Evidence suggests that a reduction in nitric oxide production related to polymorphisms of the eNOS enzyme may be linked to implantation failure and frequent miscarriages [19,23,24,25,26,27,33]. The polymorphism can affect the response of the vascular endothelium to oxidative stress and, therefore, promote vascular remodelling and cause pregnancy complications. In the present report, the polymorphic variant Asp298Asp (rs1799983) was only observed in a group of patients. There is a significant association (*p* < 0.05) between the presence of the allele and the likelihood of miscarriage. In Asian populations, there are reports associating rs1799983 with frequent miscarriage. However, in other European countries, no clear association has been found [19,25]. Even though a review by Shi and coworkers [19] mentions studies performed in admixed populations in other geographic areas, the genetic characteristics of the admixed Venezuelan population differ [30]. Even though most genes in the Venezuelan admix population are Caucasian, the African and mixed Amerindian genes differ from other Latin American populations [30]. The present report is the first on this specific population.

Although this report cannot clarify the possible associations between these two polymorphisms and RPL, control and patient selection were very strict. In addition, it included only primary RPL, which differs from several studies in which no precise classification was mentioned [45].

A combined analysis of both polymorphisms was intended to visualise the impact of the mutated alleles. Interestingly, a group of patients presented both polymorphisms, suggesting a possible association with this clinical entity. It is essential to consider the analysis of the polymorphisms of both enzymes to screen patients with RPL since it can be an interesting genetic marker to differentiate at least a subgroup of these patients. Genetic screening will provide new treatment options for these patients.

## 5. Conclusions

The mutated homozygous genotypes, SNPs rs1051740 and rs1799983, may be increased in admixed Venezuelan women with primary RPL.

## 6. Limitations of the Study

The main limitation was the number of samples. RPL samples were from patients whose medical history and follow-up lasted over two years. Most of the RPL patients were attended by the fertility clinic without success. The controls were from the same gynaecological centres. The number limitation was also due to the certification of genetic admixture for Venezuelan population only.

## Figures and Tables

**Table 1:** Demographic data from patients with RPL and controls.

	Controls	RPL
N	50	63
Age (years)	34.3 ± 6.5	36.5 ± 5
# Pregnancies (%)	1 (10%)	2 (47.6%)
	2 (60%)	3 (36.6%)
	3 (30%)	>3 (15.6%)
# Abortions (%)	0	2 (46.7%)
		>2 (53.3%)
Duration of pregnancy (weeks)	37.3 ± 2.2	7.9 ± 3.9

**Table 4:** The haplotype analysis of both rs1051740 and rs1799983 polymorphisms.

	Controls (n = 50)	Patients (n = 63)	*p*
Tyr113Tyr/Glu298Glu	34 (68%)	30 (47.6%)	0.28
Tyr113Tyr/Glu298Asp	3 (6%)	0	0.09
Tyr113Tyr/Asp298Asp	0	0	
Tyr113His/Glu298Glu	0	0	
Tyr113His/Glu298Asp	13 (26%)	23 (36.5%)	0.44
Tyr113His/Asp298Asp	0	0	
His113His/Glu298Glu	0	4 (6.3%)	0.14
His113His/Glu298Asp	1 (2%)	0	0.44
His113His/Asp298Asp	0	6 (9.5%)	**0.04**

## Data Availability

The crude data are available from the authors.
